# Selective Sorption of Noble Metals on Polymer Gel Modified with Ionic Liquid

**DOI:** 10.3390/molecules29204970

**Published:** 2024-10-21

**Authors:** Ivanka Dakova, Olga Veleva, Irina Karadjova

**Affiliations:** 1 Faculty of Chemistry and Pharmacy, University of Sofia St. Kliment Ohridski, 1 James Bourchier Blvd., 1164 Sofia, Bulgaria; ahid@chem.uni-sofia.bg; 2 Geological Institute, Bulgarian Academy of Sciences, Acad. G. Bonchev Str. BL 24, 1113 Sofia, Bulgaria; oveleva@geology.bas.bg

**Keywords:** ionic liquid modified polymer gel, noble metals, solid-phase extraction, methylimidazolium

## Abstract

The solid phase extraction of Au, Ir, Pd, Pt, and Rh on a polymer gel modified with ionic liquid containing methylimidazolium groups (MIA-PG) has been investigated. The positively charged surface of the sorbent is highly suitable for the sorption of stable chlorido complexes of the studied analytes, while the retention of base metals Cu, Fe, Ni, Zn, and Mn is negligible. Optimization experiments performed showed that, at 0.05 M HCl, the degree of sorption of Au, Ir, Pd, and Pt is above 95%, and only for Rh, the maximum degree is 65%; complete elution is achieved in the mixture of thiourea in HCl. The results obtained from the equilibrium adsorption studies are fitted in various adsorption models, such as Langmuir and Freundlich, and the model parameters have been evaluated. The kinetics analysis indicated that the adsorption of Au, Ir, Pd, Pt, and Rh onto the sorbent follows the pseudo-second-order model. Intraparticle diffusion and ion exchange reactions were the rate-limiting steps. Analytical procedures were developed for Pd, Pt, and Rh determination in road dust and soil and for Au determination in copper ore and copper concentrate. The procedures are validated by the analysis of certified reference materials. Analytical figures of merit confirmed their applicability in routine laboratory practice.

## 1. Introduction

Noble metals (NMs), six elements from the eighth group of the periodic table (Ru, Rh, Pd, Os, Ir, Pt) known as platinum group metals and Au, have been used in various industrial fields due to their attractive physical and chemical properties (electrical resistance, inertness to chemical attacks, hardness, excellent catalytic activity) [1]. These extensive applications lead to an increase in their naturally low concentration in the environment and require the development of efficient analytical procedures for both the determination of noble metals at very low background concentrations and determination of noble metals at elevated concentration levels to ensure a reliable risk assessment to ecological systems and human beings.

Sensitive instrumental methods, such as ICP-MS or ETAAS, allow for the measurement of noble metals and gold at low environmentally relevant concentrations; however, strong matrix interferences restrict their practical application for complicated samples [2]. An effective approach for their determination in “difficult” matrices, such as road dust, soils, ores, and ore concentrates, is the combination of a separation step followed by instrumental measurement. Numerous analytical techniques have been developed for the separation and enrichment of NMs, including liquid–liquid extraction [3,4,5], cloud point extraction [6,7], precipitation [8], membrane separation [9], and solid-phase extraction (SPE) [10,11,12,13,14,15,16,17]. Among them, SPE is favored because of its many benefits, such as performance simplicity, reduced amounts of organic solvents used and relatively low sample amount needed, high preconcentration factors, convenient batch procedures, clean extracts, and easy automation [18]. The correct choice of sorbent is crucial to achieve high extraction efficiency. A variety of sorbents with different characteristics, such as capacity, particle size, nature of functional groups, and matrix type (organic or inorganic), have been proposed for the SPE of NMs [10,12,13,14]. Organic polymeric materials consisting of cross-linked copolymers (solid support) and functional groups (ligands) are often used as sorbents for noble metal ions with some advantages over other sorbents, such as high adsorption capacity and selectivity, high efficiency, cost-effectiveness, fast kinetics, diverse functionality, and reusability [19,20]. Polymeric sorbents selective for precious metals often possess groups such as thiourea [21], thiazole [22], dithiocarbonate [23], cysteine [24], ethylenediamine [25], 8-aminoquinoline [26], etc. Recently, ionic liquid-based polymer gels (IL-PGs) have also found application as sorbents for the SPE of NMs due to their ion-exchange ability. Numerous studies on IL-PGs and their use for selective separation/preconcentration of NM ions have been reported: Au (III) [27,28,29,30,31], Pd (II) [32], Pt (IV) [33] and Pt (IV), Pt (II), and Pd (II) [34]. In these reports, sorbents based on different types of polymer matrix and imidazolium derivatives are proposed. According to the authors’ knowledge, until now, an ionic liquid polymer gel composed of poly(glycidyl methacrylate-co-trimethylolpropane trimethacrylate) containing 1-methylimidazolium groups (ionic liquid part) has not been used as a sorbent for the simultaneous separation and concentration of NM ions, like Au, Ir, Pd, Pt, and Rh.

In this paper, we report a study on the application of an ionic liquid modified polymer gel containing 1-methylimidazolium groups (MIA-PG), previously synthesized in our group [35], for SPE and the determination of Pd, Pt, and Rh in environmental samples (road dust and soil) and Au, Pd, and Pt in industrial samples (ore and ore concentrate). The main purpose of this research is to define the extraction efficiency of the sorbent toward Au, Ir, Pd, Pt, and Rh under optimal hydrochloric acid concentrations, contact time, and sorbent amount and to allow for the development of analytical procedures for their reliable quantification. The results obtained showed that the MIA-PG sorbent possesses high affinity and selectivity toward chlorido complexes of Au, Ir, Pd, Pt, and Rh (0.05 mol/L HCl as the sample medium) in the presence of transition metals, ensuring a fast and selective process of quantitative sorption. Adsorption isotherm and kinetics studies were used to elucidate the mechanism of the sorption process and determine the rate-controlling step. The accuracy and repeatability of the developed analytical procedures for noble metal determination based on SPE with MIA-PG confirmed their applicability in routine laboratory practice.

## 2. Results and Discussion

### 2.1. Extraction Efficiency—Optimization Studies

**Figure 1:**
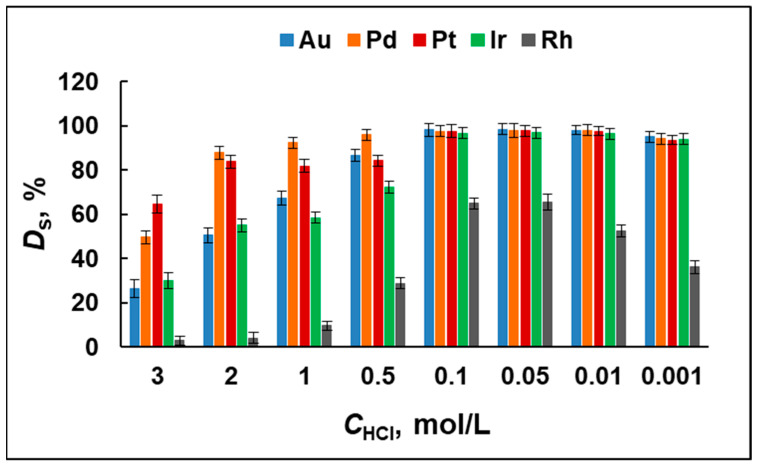
The effect of HCl concentration (*C*_HCl_) on the degree of sorption (*D*_S_) of Au, Ir, Pd, Pt, and Rh with MIA-PG (three parallel experiments).

Sorption efficiency. The synthesized sorbent MIA-PG could be regarded as a strongly basic anion exchange resin (SBAR) due to the positively charged 1-methylimidazolium functional groups on its surface. This explains the adsorption of the studied noble metals, which is strongly dependent on the behavior of their anionic chlorido-complex species. That is why the effect of HCl concentration on the degree of sorption of Au, Ir, Pd, Pt, and Rh on the MIA-PG was investigated in a wide range of HCl concentrations from 0.001 to 3.0 mol/L (Figure 1). It is clearly observed that quantitative sorption of Au, Ir, Pd, and Pt is reached at HCl concentrations of 0.01–0.1 mol/L. An increase in HCl concentration leads to a decrease in sorption efficiency. This result can be explained by the competition between chloride anions and chlorido-complex anions of the metals for positively charged sorbent binding sites. It might be expected that, at high concentrations of hydrochloric acid, the interaction with the chloride anions prevails, while at lower HCl concentrations, the interaction with the anions of the metal–chlorido complexes predominates, which ensures their quantitative sorption. The lower values of the degree of sorption in the presence of low 0.001 mol/L HCl can be explained by other reasons. The high dilution of the HCl solution is known to result in a greater complexity and diversity of chlorido complexes of metals and the increased formation of their aqua complexes [36]. Bernardis et al. [37] reported that the tendency for the metal–chlorido complexes to form ion pairs with anion-exchangers is [MCl_6_]^2−^ > [MCl_4_]^2−^ ≫ [MCl_6_]^3−^ > mixed aquachlorido complexes. This order is determined by the charge density of the species (i.e., charge-to-size ratio). Species with a low charge density are more easily paired than species with a higher charge density due to the influence of the hydrate shell size around the ions.

The results presented in Figure 1 for Rh show that quantitative sorption was not achieved in the studied range of HCl concentrations. The highest degree of sorption obtained was 65 ± 4% at 0.05–0.1 mol/L HCl. This can probably be explained by the fact that rhodium forms a three-charged anion [RhCl_6_]^3−^, characterized by a high charge density, and according to the order of propensity to interact with the counterion shown above, the weakest interaction is expected between them. The binding to the positively charged sorbent surface is further complicated by the presence of a hydrate shell. At low concentrations of HCl, Rh (III) forms negatively ([RhCl_n_(H_2_O)_6−n_]^3−n^, 0 < *n* < 6), neutrally ([Rh(H_2_O)_3_Cl_3_]), and positively ([Rh(H_2_O)_4_Cl_2_]^+^, [Rh(H_2_O)_5_Cl]^2+^) charged species, leading to a very low degree of sorption below 10% [36,38].

The results obtained undoubtedly show that, at an optimal 0.05 mol/L concentration of HCl, the proposed MIA-PG sorbent ensures quantitative sorption for Au, Ir, Pd, and Pt and approximately 65% degree of sorption for Rh. This result allows for the application of MIA-PG for the separation of all studied analytes from sample solutions using the standard addition approach or correction coefficient to compensate for the lower degree of sorption achieved for Rh.

Experiments were performed to optimize the amount of MIA-PG particles to achieve the highest degree of sorption. The results showed that, for the studied range of 10–100 mg sorbent, the enrichment was quantitative with 50, 75, and 100 mg of MIA-PG sorbent. Hence, a minimum of 50 mg of sorbent materials is sufficient to achieve the quantitative preconcentration of Au, Pd, Pt, and Ir and approximately 65% degree of sorption for Rh.

**Figure 2:**
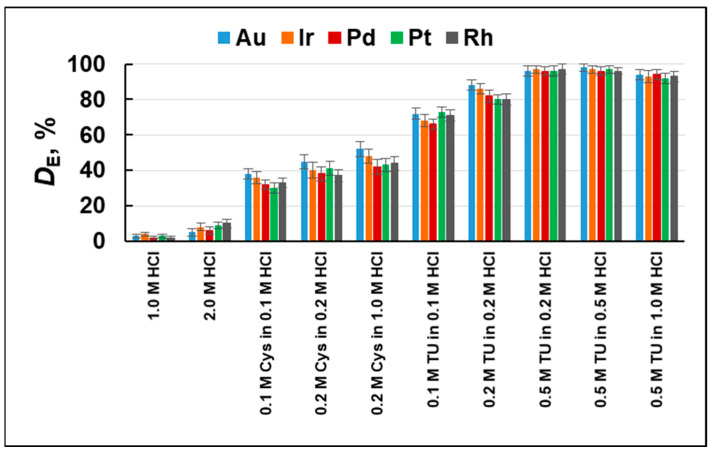
Degree of elution (*D*_E_,%) for Au, Ir, Pd, Pt, and Rh from MIA-PG using different eluents.

Desorption step. Strong basic anion exchangers are highly efficient sorbents for negatively charged chlorido complexes of platinum metals, but some problems arise with their elution due to the strong binding to the sorbent surface from acidic media. Often, the elution process is not quantitative or very slow. The experiments carried out showed that, for all of the studied analytes, the degree of elution was very low if only concentrated HCl was used (Figure 2). The reason is the formation of stable ion pairs between the anion chlorido complexes of the metals and the positively charged methylimidazolium groups of the anion exchanger. A commonly used approach for quantitative desorption in such a case is to use the competitive complex formation by using a chelating agent that leads to the decomposition of the chlorido complexes due to the formation of a more stable chelate complex. Thiourea (TU) and l-cysteine (Cys) are ligands that are well-known for their affinity for complex formation with the studied analytes due to the presence of both amine and sulfur groups that contribute to Au, Pd, Pt, Ir, and Rh binding by chelate formation [10,12]. Various concentrations of TU and Cys in the range of 0.1–0.5 mol/L mixed with HCl (0.1–1.0 mol/L) were studied in order to find the optimal eluent with the highest extraction efficiency toward all investigated noble metals. As is shown in Figure 2, the highest degree of elution was achieved when 0.5 M TU in 0.2–0.5 M HCl was used for elution. In all further experiments, the solution of 0.5 M TU in 0.2 M HCl was used for desorption of the NM chlorido complexes from the sorbent surface.

The kinetics of desorption of Au, Ir, Pd, Pt, and Rh from MIA-PG particles was investigated. It was found that complete elution is achieved for all studied NMs with 5 mL of 0.5 M TU in 0.2 M HCl for 30 min.

### 2.2. Capacity and Adsorption Isotherms

**Figure 3:**
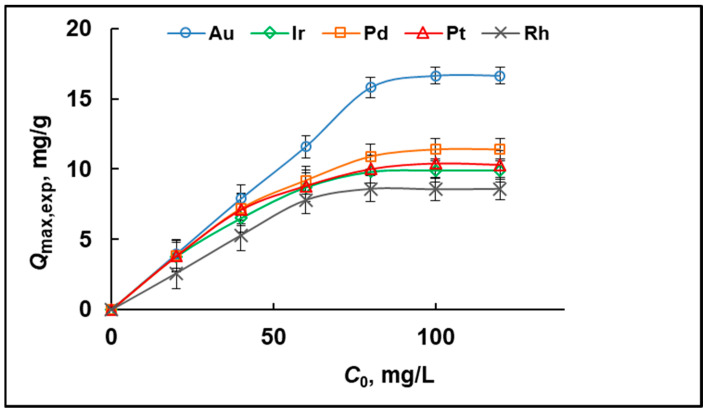
Effect of the initial concentration (*C*_0_) of Au, Ir, Pd, Pt, and Rh on the adsorption capacity (*Q*_max,exp_) of MIA-PG (0.05 mol/L HCl; dose of sorbent = 50 mg/10 mL; *C*_0_ = 2 μg/L; temperature 25 °C).

The maximal experimental adsorption capacities (*Q*_max,exp_) of MIA-PG towards Au, Ir, Pd, Pt, and Rh were determined after saturation of the sorbent with the NM ions under optimum conditions at a temperature of 25 °C. For this purpose, increasing amounts of Au, Ir, Pd, Pt, or Rh (*C*_0_ = 20–120 mg/L) were added to 50 mg of sorbent particles, and their equilibrium concentration after adsorption was measured by ICP-OES. As shown in Figure 3, the amount of all studied ions adsorbed per unit mass of sorbent increased with their initial concentration and reached plateau values, determining the adsorption capacity values. The determined *Q*_max,exp_ of MIA-PG towards Au, Ir, Pd, Pt, and Rh is given in Table 1.

The equilibrium experimental data were analyzed by the Langmuir and Freundlich adsorption isotherm models, which are expressed by the following equations:

Langmuir isotherm model:

(1) $$\frac{C_{e}}{Q_{e}}=\frac{C_{e}}{Q_{\max}}+\frac{1}{b\cdot Q_{\max}}$$

Freundlich isotherm model:

(2) $$\ln Q_{e}=\ln k_{F}+n^{-1}\cdot \ln C_{e}$$

where *C*_e_ (mg/L) and *Q*_e_ (mg/g) are the equilibrium concentration of analytes in the solution and the adsorption capacity of MIA-PG at equilibrium; *Q*_max_ (mg/g) is the calculated maximum adsorption capacity; *b* (L/mg) is the Langmuir constant; and *k*_F_ and *n* are Freundlich constants incorporating all factors that affect the adsorption process such as capacity and intensity. The Langmuir model assumes monolayer sorption on a surface with a finite number of identical sites [39]. The Freundlich model is based on the assumption that the adsorbent has a heterogeneous surface composed of adsorption sites with different energies and affinities [40].

The results obtained (Figure S1, Table 1) showed that the experimental data are better modelled by the Langmuir isotherm model because *R*^2^ (Langmuir) > *R*^2^ (Freundlich). This result may be interpreted as proof that the analytes’ sorption process occurs on a uniform binding site, forming a surface monolayer. The validity of this conclusion is further supported by the comparable values between the experimentally obtained (*Q*_max,exp_) and the estimated values (*Q*_max_) from the Langmuir isotherm model. The Langmuir adsorption capacity achieved is lower than the published values for sorbents based on ionic liquid. However, the objective of our study is selective retention of low, environmentally relevant concentrations of noble metals. From such a point of view, the selectivity of the sorbent is a much more important parameter than adsorption capacity.

### 2.3. Adsorption Kinetics Studies

**Figure 4:**
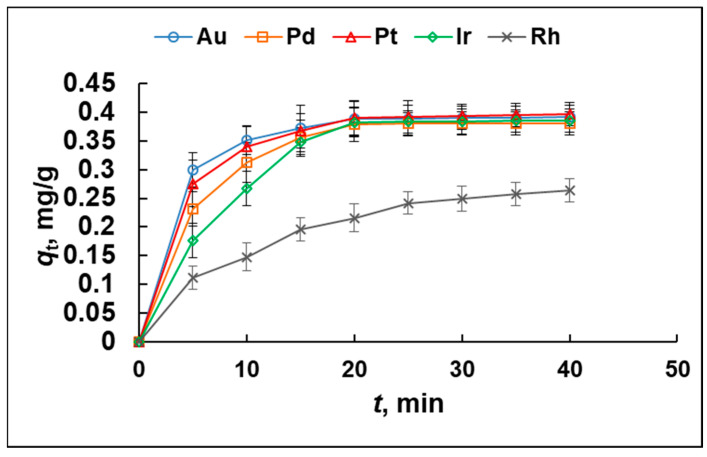
Adsorption kinetics plots of MIA-PG towards Au, Ir, Pd, Pt, and Rh, *q*_t_—amounts of Au, Ir, Pd, Pt, and Rh ions retained per mass unit of sorbent at time *t*: (0.05 mol/L HCl); dose of sorbent/sample volume = 50 mg/10 mL; *C*_0_ = 2 μg/L; temperature 25 °C).

Adsorption kinetics experiments were performed (Section 3.3.4.) to assess the effect of contact time on the sorbent binding capacity. According to the results (Figure 4), the saturation values of the adsorption capacity of MIA-PG towards Au, Ir, Pd, Pt, and Rh are reached within 25 min, so the analytes’ adsorption process is sufficiently quick for practical applications.

The experimental data collected from the adsorption kinetic tests were fitted by pseudo-first-order (PFO) and pseudo-second-order (PSO) kinetics models in order to determine the controlling mechanism of the adsorption process. The PFO model postulates that the rate of adsorption site occupancy is proportional to the number of vacant sites [41], while the PSO model is based on the assumption that the adsorption rate is controlled by the chemisorption mechanism [42]. For these models, the linear form of the equations looks like this:

pseudo-first-order model:

ln(*q_e_* − *q_t_*) = ln*q_e_* − *k*_1_·*t* (3)

pseudo-second-order model:

(4) $$\frac{t}{q_{t}}=\frac{1}{k_{1}\cdot q_{e}^{2}}+\frac{t}{q_{e}}$$

where *q*_e_, *q*_t_ are the amounts of Au, Ir, Pd, Pt, and Rh ions retained per mass unit of sorbent at equilibrium and at time *t*, (mg/g), respectively; and *k*_1_, *k*_2_ are the rate constants of the pseudo-first-order kinetics model (1/min) and pseudo-second-order kinetics model (g/mg∙min), respectively.

The linear plots of the pseudo-first-order and pseudo-second-order models for Au, Ir, Pd, Pt, and Rh ions sorption onto MIA-PG are shown in Figure S2. The corresponding kinetics parameters and the correlation coefficients are summarized in Table 2. It can be observed that the calculated values of *q*_e,calc_ from the pseudo-second-order equation are in good agreement with the experimental data (*q*_e,exp_). The correlation coefficients *R*^2^ from the pseudo-second-order model are higher than those from the pseudo-first-order model. These results prove that the rate-limiting step is the ion exchange of Au, Ir, Pd, Pt, and Rh (III) ions onto polymer particles, thus confirming the strong interactions of methylimidazolium fragments in MIA-PG with the studied NM ions.

Analysis of rate-limiting step. The chemical structure of MIA-PG shows that the studied material is a strongly basic anion exchanger, which predicts that the retention of the chlorido complexes of Au, Ir, Pd, Pt, and Rh on the sorbent is due to the ion exchange mechanism. The adsorption of the analytes on the MIA-PG can be explained by sequential processes: (i) external diffusion of ions through the liquid film surrounding the particle; (ii) internal diffusion of ions through the polymeric matrix; and (iii) chemical reaction with the sorbent functional groups. One of these steps is usually the slowest, so it can be considered the rate-determining step of the process. Firstly, to assess which step is rate-determining for the adsorption of NMs onto MIA-PG, a shell-progressive model is applied. This model is based on the fluid-particle chemical reaction and is assumed to proceed first on the outer surface of the particle [43]. It describes the relationship between contact time (*t*) and adsorption degree (*X* = *q*_t_/*q*_e_) and is represented by the following three equations:

External diffusion is controlling process:

(5) $$X=\frac{3c_{A}k_{F}}{a_{s}{rc}_{s}}t$$

Internal diffusion is controlling process:

(6) $$[3-3(1-{X)}^{\frac{2}{3}}-2X]=\frac{6D_{e}c_{A}}{a_{s}r^{2}c_{s}}t$$

Chemical reaction is controlling process:

(7) $$[1-(1-{X)}^{\frac{1}{3}}]=\frac{k_{s}c_{A}}{r}t$$

where *c*_A_ (mol/L) is the concentration of NM ions in solution, *c*_s_ (mol/L) is the concentration of NM ions that remained in solution after the sorption process, *k*_F_ (m/s) is the mass transfer coefficient of species through the liquid film, *D*_e_ (m^2^/s) is the diffusion coefficient through the reacted layer in the solid phase, *k*_s_ (m/s) is the reaction constant based on the surface, and *r* (m) and *a*_s_ are the average radius of the adsorbent particles and the stoichiometric coefficient, respectively.

The experimental kinetics data were fitted by the shell-progressive model to assess which step controls the adsorption process. The results obtained are shown in Table 3. It was found that, among the three controlling mechanisms, the correlation coefficients (*R*^2^) obtained for internal diffusion have the highest values. This indicates that the rate-limiting step is migration of the Au, Ir, Pd, Pt, and Rh chlorido complexes inside the sorbent particles (internal diffusion).

To confirm that the internal diffusion step is the controlling step of the adsorption process, the experimental data were fitted by the intra-particle diffusion model (Weber–Morris model). This model can be represented by the following equation [44]:

(8) $$q_{t}=k_{\mathrm{diff}}\cdot t^{1/2}+C$$

where *k*_diff_ (mg/g∙min^1/2^) is the intra-particle diffusion rate constant and intercept C, obtained by extrapolation of the linear portion of the plot of *q*_t_ versus *t*^1/2^, is an indicator to express the boundary layer thickness.

The graph of *q*_t_ versus *t*^1/2^ (Figure S3) demonstrates the presence of two distinct line parts with different slopes, providing evidence for the involvement of multiple steps in the adsorption process. The first region can be explained by the mass transfer of the NM chlorido complexes from the bulk solution to the adsorption surface, whereas the second region can be related to their internal diffusion into the particle cavities. The results in Table 3 indicate that the first step has a higher adsorption rate than the second. The fact that the boundary layer thickness values (C) deviate from zero suggests that surface adsorption (specifically ion exchange) is responsible for the retention of Au, Ir, Pd, Pt, and Rh chlorido complexes on the MIA-PG. Most probably, both pore diffusion and the mass transfer resistance in the external liquid film regulate this process [45].

### 2.4. Analytical Application

#### 2.4.1. Selectivity Studies

The most serious problem for the accurate determination of NMs in complicated matrices, such as soils, road dust, soil, ores, and ore concentrates, by ICP-MS is not the instrumental sensitivity but the many interferences due to the formation of oxides and polyatomic and double-charged ions in the presence of matrix constituents. The mass resolution required for their elimination is not available in common quadrupole mass spectrometers. That is why the main purpose of the analytical application of the synthesized sorbent MIA-PG is the efficient separation of noble metals with high selectivity from interfering elements. As a first step, the selectivity of MIA-PG toward base metals, usual constituents of environmental and industrial samples, was studied. The results obtained following the procedure described in Section 3.3.4 are shown in Table 4.

From these results, it is clear that even high levels of the coexisting base metals do not affect the recovery of Pd, Pt, Ir, and Au ions at the optimal HCl concentration. The best selectivity was achieved at 0.05 mol/L HCl, the lowest possible for quantitative sorption of noble metals. The degree of sorption for Rh was almost the same as from an aqueous solution, showing high selectivity of MIA-PG toward Rh in the presence of base metals. It might be concluded that MIA-PG is a highly suitable and promising sorbent for the recovery of noble metals from samples with a complicated matrix, in this way ensuring their interference-free measurement by ICP-MS.

#### 2.4.2. Determination of Pd, Pt, and Rh in Road Dust and Soils

An analytical procedure was developed for noble metal determination in road dust and soils. Digestion was carried out with a mix of HCl + HNO_3_ + HF under an optimized MW temperature program. After MW digestion, the sample solution was subjected to an additional evaporation step in order to remove the acid mixture and ensure a 0.05 mol/L final concentration of HCl before the sorption step. Two parallel samples were used, and to one of them, an additional spike of a mixture of Au, Ir, Pd, Pt, and Rh (0.5 µg/L) was added to examine the exact degree of sorption of the studied analytes and recalculate the results based on the standard addition calibration method. In this way, a lower degree of Rh sorption would not affect the final result. The developed procedure was applied to CRM OREAS 45d (soil) and to CRM BCR-723 Platinum and Rhodium in road dust, and in Table S1, the results achieved for the analyte recovery and calculated results for the certified elements are presented.

The results showed recoveries for all the studied analytes above 95%. Very good agreement was found between the certified values and the concentrations determined following the developed analytical procedure (Students t-criterion), thus confirming its validity and versatility. The relative standard deviations varied between 4 and 10%. The calculated limit of quantification (10 σ criterion) based on the purity of the acids used is 5 ppb for all analytes (0.5 µg/L is accepted as the LOQ for ICP-MS measurement). The developed procedure is suitable for the analysis of environmental samples of soil and road dust and, for sure, might also be applied to sediment analysis.

#### 2.4.3. Determination of Au, Pd, and Pt in Polymetallic Ore and Copper Concentrate

Bulgaria is a producer of copper concentrate from copper ore, and one of the biggest smelters for copper production is situated in Pirdop, Bulgaria. Determination of Au, Pt, and Pd is an important analytical task. An analytical procedure was developed for Au, Pd, and Pt determination in copper concentrate. Digestion was carried out with a mix of HCl + HNO_3_ (aqua regia) under an optimized MW temperature program. In this way, the sample was dissolved partially; however, the preliminary results confirmed that Au, Pd, and Pt were quantitatively extracted from the sample solution. In order to check the recoveries achieved for the studied analytes, a developed analytical procedure was applied to the CRM OREAS 13b and OREAS 505d. The calculated results for the certified elements as well as the recoveries achieved are presented in Table S2.

As can be observed, the developed analytical procedure is characterized by very good accuracy. A comparison of the obtained results with the certified values does not show a statistically significant difference (Student t-criterion). The relative standard deviations varied between 5 and 10%. The calculated limit of quantification (10 σ criterion) based on the purity of the acids used is 5 ppb for all analytes (0.5 µg/L is accepted as the LOQ for ICP-MS measurement). The procedure is simple and easy to apply, suitable for laboratory practice in mining and industrial laboratories.

In addition, model experiments were carried out to assess the repeatability of the sorbent synthesis procedure. The results for the degree of sorption/degree of elution obtained for the studied analytes with sorbents prepared from different batches showed values statistically close to those presented in Table 4, in this way confirming high repeatability of the synthesis procedure. The extraction efficiency of the sorbent after several sorption/desorption cycles was tested in the frame of the whole study, and it was found that, after 20 sorption/desorption cycles, the degree of sorption statistically dropped down.

## 3. Experimental

### 3.1. Apparatus

The concentrations of NMs in the model experiments investigating the extraction efficiency of the MIA-PG sorbent were measured by an inductively coupled plasma optical emission spectrometer (ICP-OES, Horiba Jobin Yvon Ultima 2, Longjumeau Cedex, France). All measurements were carried out with at least three replicates. The concentration of Pd, Pt, Rh, and Au during the development of the analytical procedures and their application to the certified reference materials was measured by an inductively coupled plasma mass spectrometer “X SERIES 2”—Thermo Scientific, Waltham, MA, USA with a 3-channel peristaltic pump; concentric nebulizer; Peltier-cooled spray chamber (4 °C); Xt interface option; and Ni cones. Optimized instrumental parameters were the following: forward plasma power of 1400 W; plasma gas flow 13 L/min; nebulizer flow 0.85 L/min; dwell time 30 ms; measurements 3 × 30 scans.

An EBA 20× *g* centrifuge (DJB Labcare Ltd., Newport Pagnell, England) was used to separate polymer particles and the solution containing NMs in the batch experiments. A universal orbital shaker OS-20 (Boeco, Hamburg, Germany) was used for the adsorption/desorption experiments.

### 3.2. Reagents and Materials

All reagents and solvents used were of analytical-reagent grade. The stock standard solutions 100 mg/L each of Ru, Rh, Pd, Os, Ir, Pt, and Au in 10% hydrochloric acid (TraceCERT^®^) were used (Sigma-Aldrich, Steinheim, Germany). The stock standard solutions for Cd (II), Cu (II), Co (II), Fe (III), Ni (II), Pb (II), and Zn (II) (1000 μg/mL) were Titrisol, Merck (Darmstadt, Germany) in 2% HNO_3_. The working aqueous standard solutions were prepared daily by appropriate dilution with doubly distilled water. The elution solutions were prepared from nitric acid, hydrochloric acid, l-cysteine (Cys), and thiourea (TU) (Merck, Darmstadt, Germany). All acids used in the sorption, elution, and digestion procedures are Suprapur (Merck, Darmstadt Germany).

Certified reference material BCR-723 Platinum and Rhodium in road dust; Certified reference material OREAS 45d, Ferruginous soil; Certified reference material OREAS 13d, gabbronorite (prepared from ores of platinum group elements (PGEs), copper, nickel, and gold dispersed in a gabbro matrix), Certified reference material OREAS 505d (porphyry Cu-Au certified reference material prepared from a blend of ores, barren granodiorite, and minor additions of copper and molybdenum concentrates): Au 544 (535–553) ppb, Aqua Regia Digestion.

The reagents used to prepare the MIA-PG sorbent were glycidyl methacrylate, trimethylolpropane trimethacrylate, 2,2′-azobisisobutyronitrile, 1-methylimidazole (MIA) (Merck, Darmstadt, Germany), and acetonitrile (ACN) (Labscan, Dublin, Ireland). The mean diameter of the obtained spherical particles is 2.34 µm. The specific surface area, total pore volume, and average pore diameter are 27 m^2^/g, 0.10 m^3^/g, and 15 nm, respectively [35].

### 3.3. Procedures

#### 3.3.1. Sorbent Synthesis

The sorbent MIA-PG was prepared following the synthesis procure described in detail in [35]. Briefly, poly(glycidyl methacrylate-co-trimethylolpropane trimethacrylate) was synthesized via precipitation copolymerization of glycidyl methacrylate (0.546 mmol), trimethylolpropane trimethacrylate (0.894 mmol), and AIBN (32 mg) in acetonitrile (25 mL). In the next step, MIA (6.3 mmol) and NaOH (0.1 g) were dissolved in a mixture of ethanol (20 mL) and H_2_O (25 mL) in a flask, and the polymer particles obtained (0.5 g) were added. The suspension was stirred and refluxed at 80 °C for 12 h.

#### 3.3.2. Batch Sorption Procedure

The sorption efficiency of the MIA-PG toward chlorido complexes of Au, Ir, Pd, Pt, and Rh was studied in a batch mode. The influence of HCl concentration on the degree of sorption of the analytes was investigated in the range 0.001–3 mol/L. Aqueous solutions of HCl solution with the desired concentration were mixed with 2 µg/mL of Au, Ir, Pd, Pt, and Rh and 50 mg of MIA-PG and shaken for 30 min. Samples were centrifuged (5800 rpm) for 10 min, supernatants were removed, and the concentrations of the analytes were measured by ICP-OES. Degrees of sorption (*D*_S%_) were calculated as follows:

(9) $$D_{S}\left(\%\right)=\frac{A_{i}-A_{eff}}{A_{i}}\times 100,$$

where *A*_eff_ (µg) is the amount of the analyte in the supernatant solution and *A*_i_ (µg) is the initial amount of Au, Ir, Pd, Pt, and Rh added.

#### 3.3.3. Desorption Studies

Several mixtures of HCl acid and thiourea at different concentration levels were tested as potential eluents. All experiments were performed after loading the sorbent with 2 µg Au, Ir, Pd, Pt, and Rh under optimal conditions, followed by treatment with 5.0 mL of each eluent for 30 min on an electrical shaker. Degree of elution (*D*_E%_) was defined as follows:

(10) $$D_{E}\left(\%\right)=\frac{A_{el}}{A_{i}-A_{eff}}\times 100,\;\;$$

where *A*_el_ (µg) is the amount of Au, Ir, Pd, Pt, and Rh in the eluate.

#### 3.3.4. Kinetics Studies

The kinetics studies were carried out by the following procedure: a total of 10 mL of 0.05 mol/L HCl was mixed with 2 µg/mL of Au, Ir, Pd, Pt, and Rh and 50 mg of MIA-PG. The samples were shaken vigorously for time intervals of 5, 10, 15, 20, 25, 30, 35, and 40 min at a temperature of 25 °C. The supernatants were removed after centrifugation, and the concentrations of the analytes were measured by ICP-OES.

#### 3.3.5. Selectivity Studies

Model solutions containing 1000 µg/L Fe, Cu, Mn, Zn, Ni, Cd, and Pb were prepared in 0.05 mol/L HCl; spiked with 100 µg/L Au, Ir, Pd, Pt, and Rh; and mixed with 50 mg of MIA-PG. The solutions were shaken for 30 min and centrifuged at 5000 rpm. The supernatant above the sorbent was removed, and all elements were measured by ICP-OES. The MIA-PG particles were washed with distilled water and shaken with a mixture of 5 mL 0.5 M TU in 0.2 M HCl for 30 min. After centrifugation, the elements Au, Ir, Pd, Pt, and Rh were measured in the eluate by ICP-OES. The degree of sorption and elution was calculated using Equations (9) and (10).

#### 3.3.6. Procedure for Pd, Pt, and Rh Determination in Road Dust and Soils

A sample of approximately 500 mg of dust or soil was weighed directly in MW high-pressure teflon vessels, 6 mL of conc. HCl, 2 mL of conc. HNO_3_, and 2 mL of conc. HF was added, and the samples were left to stay overnight. The MW digestion program for both dust and soil matrices included three steps: 1. 250 W (3 min); 2. 400 W (5 min); 3. 600 W (10 min). At the end of the digestion program, the solution was evaporated almost to dryness on a hot plate. The residue was dissolved in 5 mL of 0.5 M HCl, transferred to a 25 mL volumetric flask, and diluted up to the mark with doubly distilled water. Two parallel samples of 10 mL were transferred into centrifuge tubes. One of these samples was spiked with 1 µg/L Au, Ir, Pd, Pt, and Rh. Approximately 50 mg sorbent MIA-PG was added, and both samples were shaken for 30 min. After centrifugation, the supernatant was removed, sorbent washed with doubly distilled water, and elution carried out with 5 mL of 0.5 M TU in 0.2 M HCl. After centrifugation, Au, Ir, Pd, Pt, and Rh were measured in the supernatant by ICP-MS under optimal instrumental parameters.

#### 3.3.7. Procedure for Au Determination in Cooper Concentrate

A sample of approximately 500 mg of copper concentrate was weighed in high-pressure Teflon vessels; a total of 7.5 mL of conc. HCl and 2.5 mL of conc. HNO_3_ were added, and the samples were left to stay overnight. The MW digestion program for copper concentrate consists of 2 steps: 1. 250 W (10 min); 2. 600 W (15 min). At the end of the digestion program, the solution was evaporated almost to dryness on a hot plate. The residue was dissolved in 5 mL of 0.5 mol/L HCl, transferred to a 25 mL volumetric flask, and diluted up to the mark with doubly distilled water. Two parallel samples of 10 mL were filtered and transferred into a centrifuge tube. Approximately 50 mg sorbent MIA-PG was added, and both samples were shaken for 30 min. After centrifugation, the supernatant was removed, sorbent washed with doubly distilled water, and elution carried out with 5 mL of 0.5 M TU in 0.2 M HCl. After centrifugation, Au, Pd, and Pt were measured in the supernatant by ICP-MS under optimal instrumental parameters.

## 4. Conclusions

The ionic liquid modified polymer gel containing methylimidazolium groups (MIA-PG) was found useful for the selective extraction of precious metals from complicated matrices, such as road dust, soil, and copper ores and copper concentrate. The optimization experiments showed that, at 0.05 M HCl, the positively charged surface of the sorbent allowed for the retention of chlorido complexes of Au, Ir, Pd, Pt, and Rh, while the sorption of base metals was negligible. The solution of 0.5 M thiourea in 0.2 M HCl can effectively desorb the precious metal ions from PG-MIA. The adsorption–desorption cycle results demonstrated that the MIA-PG could be reused up to 20 times without a significant change in the amount of adsorption for the studied metal ions. The experimental data were well fit by the Langmuir isotherm model, and the adsorption coefficients agreed well with the conditions supporting favorable adsorption. The kinetic studies indicated that the contact time for sorption/desorption was suitable for the MIA-PG application in laboratory practice. The regression results of the intra-particle diffusion model suggested that intraparticle diffusion was not the only rate-controlling step. Therefore, there are good prospects for MIA-PG in practical applications for the determination of Au, Ir, Pd, Pt, and Rh in environmental and industrial samples. In this aspect, analytical procedures were developed and applied for the analysis of CRM (road dust, soil, ore, and copper ore concentrate). Very good agreement was achieved for the certified values, as well as the calculated LOQ and RSD confirmed the validity and applicability of the proposed procedures.

## Figures and Tables

**Table 1:** Experimental and fitting parameters of the various isotherm models for adsorption of Au, Ir, Pd, Pt, and Rh ions onto the MIA-PG at a temperature of 25 °C (dose of sorbent/sample volume = 50 mg/10 mL; *C*_0_ = 2 μg/mL; 0.05 mol/L HCl; temperature 25 °C).

Adsorption Isotherm Model	Parameters	Au	Ir	Pd	Pt	Rh
Experimental adsorption capacity	*Q*_max,exp_ (mg/g)	16.64	9.90	11.40	10.30	8.60
Langmuir	*Q*_max_ (mg/g)	16.37	10.19	11.95	10.72	8.33
	*b* (L/mg)	0.77	0.88	0.36	0.43	0.08
	*R* ^2^	0.9936	0.9997	0.9989	0.9993	0.9949
Freundlich	*k*_F_	7.42	5.03	4.33	4.31	1.44
	*n*	4.22	5.30	3.80	4.29	2.61
	*R* ^2^	0.8736	0.6870	0.9327	0.9232	0.8952

**Table 2:** Parameters of pseudo-first-order and pseudo-second-order models for adsorption of Au, Ir, Pd, Pt, and Rh ions onto the MIA-PG (dose of sorbent/sample volume = 50 mg/10 mL; *C*_0_ = 2 μg/mL; 0.05 mol/L HCl; temperature 25 °C).

Model	Parameters	Au	Ir	Pd	Pt	Rh
Pseudo-first-order model	*q_e_*_,*exp*_ (mg/g)	0.39	0.40	0.38	0.40	0.26
	*q_e_*_,*calc*_ (mg/g)	0.1107	0.2066	0.2052	0.1837	0.5878
	*k*_1_ (1/min)	0.1159	0.0876	0.156	0.1274	0.1474
	*R* ^2^	0.9168	0.8040	0.8814	0.9645	0.8667
Pseudo-second-order model	*q_e_*_,*calc*_ (mg/g)	0.4097	0.4597	0.4174	0.4227	0.3411
	*k*_2_ (1/min)	1.5897	0.3514	0.7822	1.0388	0.2264
	*R* ^2^	0.9996	0.9858	0.9968	0.9992	0.9945

**Table 3:** Parameters of shell-progressive and intra-particle diffusion models for adsorption of Au, Ir, Pd, Pt, and Rh ions onto the MIA-PG (dose of sorbent/sample volume = 50 mg/10 mL; *C*_0_ = 2 μg/mL; 0.05 mol/L HCl; temperature 25 °C).

Model	Parameters	Au	Ir	Pd	Pt	Rh
Intra-particle diffusion model, Region 1	*k_diff_* (mg/g min^1/2^)	0.0327	0.0794	0.0550	0.0426	0.0139
	C (mg/g)	0.2380	0.0139	0.1258	0.1936	0.0794
	*R* ^2^	0.9179	0.9401	0.9214	0.9304	0.9906
Intra-particle diffusion model, Region 2	*k_diff_* (mg/g min^1/2^)	0.0018	0.0018	0.0009	0.0036	0.3741
	C (mg/g)	0.3805	0.3741	0.3755	0.3738	0.0018
	*R* ^2^	0.9988	0.9988	0.9988	0.9988	0.9973
Shell-progressive model, external diffusion	*k*_F_ (µm/s)	7.0 × 10^−7^	2.2 × 10^−6^	2.5 × 10^−6^	3.1 × 10^−6^	3.3 × 10^−6^
	*R* ^2^	0.6561	0.6728	0.6353	0.6834	0.8972
Shell-progressive model, internal diffusion	*D*_e_ (µm^2^/s)	1.2 × 10^−6^	2.3 × 10^−6^	3.0 × 10^−6^	4.7 × 10^−6^	3.1 × 10^−6^
	*R* ^2^	0.8025	0.7941	0.7763	0.8505	0.9938
Shell-progressive model, chemical reaction	*K*_s_ (µm/s)	2.1 × 10^−2^	2.4 × 10^−2^	1.6 × 10^−2^	2.5 × 10^−2^	1.8 × 10^−2^
	*R* ^2^	0.7927	0.7178	0.7561	0.8469	0.9666

**Table 4:** Selectivity studies—degree of sorption *D*_S_ (%) (mean ± SD) of Au, Ir, Pd, Pt, and Rh in the presence 1000 µg/L base metals (three parallel experiments) and degree of elution *D*_E_ (%) (mean ± SD).

Element	Degree of Sorption, *D*_S_ (%)	Degree of Elution, *D*_E_ (%)
Au	97 ± 2	97 ± 2
Ir	96 ± 2	96 ± 2
Pd	98 ± 1	98 ± 1
Pt	97 ± 2	97 ± 2
Rh	65 ± 3	97 ± 3
Cd	<1	
Cu	<1	
Fe	<3	
Pb	<5	
Co	<1	
Ni	<1	

## Data Availability

Data are contained within the article and Supplementary Materials.

## Supplementary Materials

The following supporting information can be downloaded at: https://www.mdpi.com/article/10.3390/molecules29204970/s1, Figure S1: Langmuir (a), Freundlich (b) isotherms for adsorption of Au, Ir, Pd, Pt, and Rh on the MIA-PG.; Figure S2: The linear fitting curves of pseudo-first-order reaction (a) and pseudo-second-order reaction (b) for MIA-PG (dose of sorbent/sample volume = 50 mg/10 mL; *C*_0_ = 2 μg/mL Au, Ir, Pd, Pt, and Rh; 0.05 mol/L HCl; temperature 25 °C); Figure S3: The linear fitting curves of the intra-particle diffusion model for MIA-PG (dose of sorbent/sample volume = 50 mg/10 mL; *C*_0_ = 2 μg/mL Au, Ir, Pd, Pt, and Rh; 0.05 mol/L HCl; temperature 25 °C); Table S1: Recoveries achieved for studied elements and results for certified values (presented as in the certificate), (three parallel samples); Table S2: Recoveries achieved for studied elements and results for certified values (presented as in the certificate), (three parallel samples).
