# Supplementary material for: Selective Sorption of Noble Metals on Polymer Gel Modified with Ionic Liquid
Source: Molecules. 2024 Oct 21;29(20):4970. doi: 10.3390/molecules29204970 (PMC11510461; doi:10.3390/molecules29204970)
Supplement: Supplementary file 1 [file molecules-29-04970-s001.zip › molecules-3255989-supplementary.pdf]

Supplementary Materials

Selective Sorption of Noble Metals on Polymer Gel Modified with Ionic Liquid

Ivanka Dakova [1], Olga Vleva [2] and Irina Karadjova [1*]

[1] Faculty of Chemistry and Pharmacy, University of Sofia “St. Kliment Ohridski”, 1, James. Bourchier Blvd.1, 1164 Sofia, Bulgaria; ahid@chem.uni-sofia.bg
2 Geological Institute, Bulgarian Academy of Sciences, 1113 Sofia, Acad. G. Bonchev Str. BL 24, Bulgaria; oveleva@geology.bas.bg
* Correspondence: karadjova@chem.uni-sofia.bg

Figures

Section: *2.2. Capacity and adsorption isotherms*

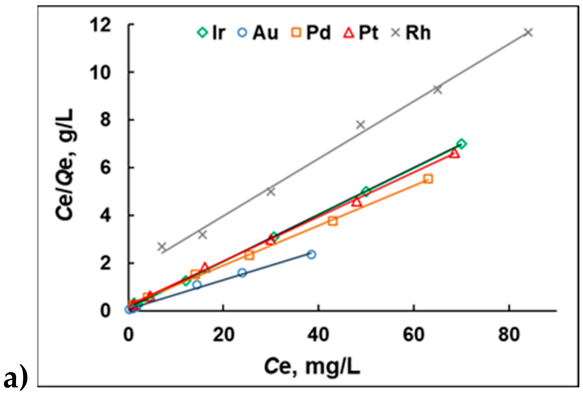

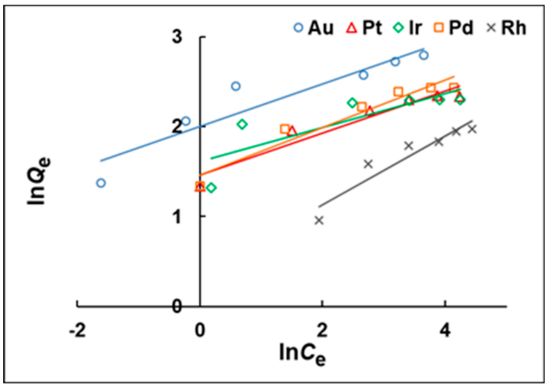

Figure S1. Langmuir (a), Freundlich (b) isotherms for adsorption of Au, Ir, Pd, Pt, and Rh on the MIA-PG.

Section: *2.3. Adsorption kinetics studies*

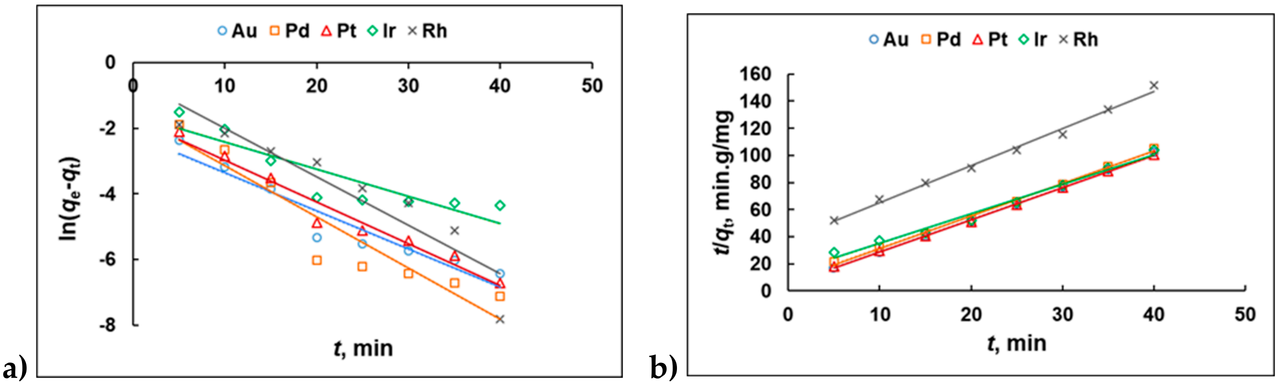

Figure S2. The linear fitting curves of pseudo-first-order reaction (a) and pseudo-second-order reaction (b) for MIA-PG (sorbent dose = 50 mg/10 mL; c_0 = 2 μg/mL Au, Ir, Pd, Pt, and Rh, 0.05 mol/L HCl; temperature 25 °C).

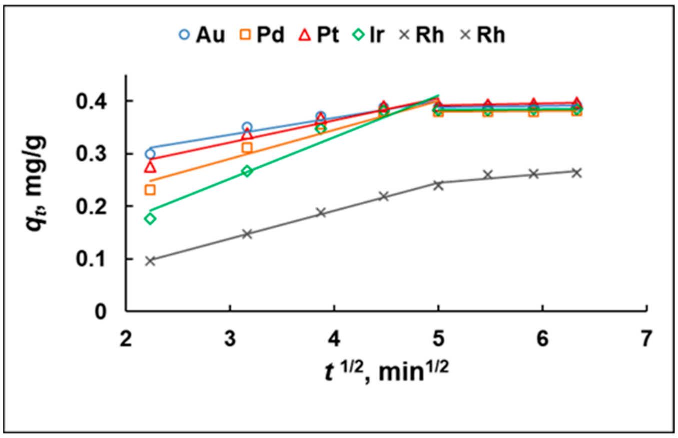

Figure S3. The linear fitting curves of the intra-particle diffusion model for MIA-PG (sorbent dose = 50 mg/10 mL; c_0 = 2 μg/mL Au, Ir, Pd, Pt, and Rh, 0.05 mol/L HCl; temperature 25 °C).

Tables

Section: *2.4. Analytical Application*

Table S1. Recoveries achieved for studied elements and results for certified values (presented as in the certificate), (three parallel samples).

Sample	Element	Recovery, (%)	Certified values (Pb fire assay)	Results Proposed procedure
CRM OREAS 45d (soil)			Mean, ppb (sd)	Mean, ppb (sd)
	Au	104±2	23 (2)	24 (2)
	Pd	101±2	34.6 (2.3)	35 (3)
	Pt	95±1	48.5 (3.1)	46 (4)
CRM BCR-723 (road dust)			mean, µg/kg (U)	Mean, µg/kg (U)
	Pd	97±2	6.1 (1.9)	5.9 (0.5)
	Pt	102±3	81.3 (2.5)	83 (3)
	Rh	95±4*	12.8 (1.3)	12.1 (2)

* - Calculated against standard addition

Table S2. Recoveries achieved for studied elements and results for certified values (presented as in the certificate), (three parallel samples).

Sample	Element	Recovery, (%)	Certified values (Pb fire assay)	Results Proposed procedure
CRM OREAS 13b			Mean, ppb (sd)	Mean, ppb (sd)
	Au	98±2	211 (13)	206 (15)
	Pd	95±3	131 (9)	125 (11)
	Pt	96±2	197 (13)	189 (14)
CRM BCR-723 (road dust)			Mean, ppm (sd) Aqua regia	Mean, ppm (sd) Aqua regia
	Au	101±2	0.552 (0.023)	0.556 (0.028)
